# Supplementary material for: Organellar Genomes of Sargassum hemiphyllum var. chinense Provide Insight into the Characteristics of Phaeophyceae
Source: Int J Mol Sci. 2024 Aug 6;25(16):8584. doi: 10.3390/ijms25168584 (PMC11354929; doi:10.3390/ijms25168584)
Supplement: Supplementary file 1 [file ijms-25-08584-s001.zip › Figure S1. Secondary structures of tRNAs of Sargassum hemiphyllum var. chinense.pdf]

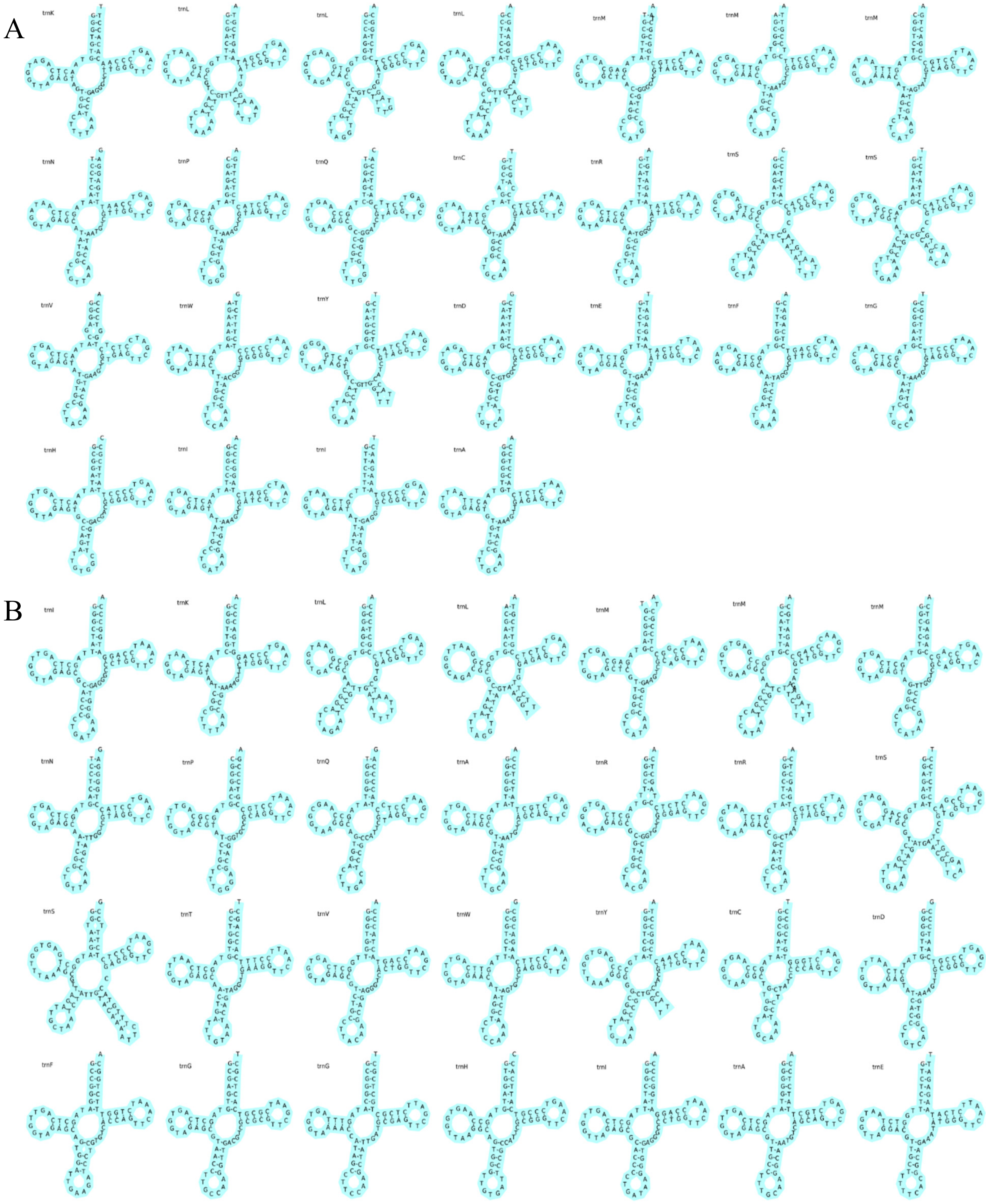

Figure S1. Secondary structures of tRNAs of *Sargassum hemiphyllum* var. *chinense* mtDNAs (**A**) and cpDNAs (**B**). Each region of tRNA is named as follows: Amino acid accepting stem, AAS (upper arm); dihydrouridine stem and loop, DSL (left arm); anticodon stem and loop, ASL (lower arm); thymidine stem and loop, TSL (right arm); variable stem and loop, VSL (between ASL and TSL).
