# Supplementary material for: Organellar Genomes of Sargassum hemiphyllum var. chinense Provide Insight into the Characteristics of Phaeophyceae
Source: Int J Mol Sci. 2024 Aug 6;25(16):8584. doi: 10.3390/ijms25168584 (PMC11354929; doi:10.3390/ijms25168584)
Supplement: Supplementary file 1 [file ijms-25-08584-s001.zip › Figure S4. PR2-plot analysis of mtDNA and cpDNA in Fucales, Ectocarpales and Laminariales.pdf]

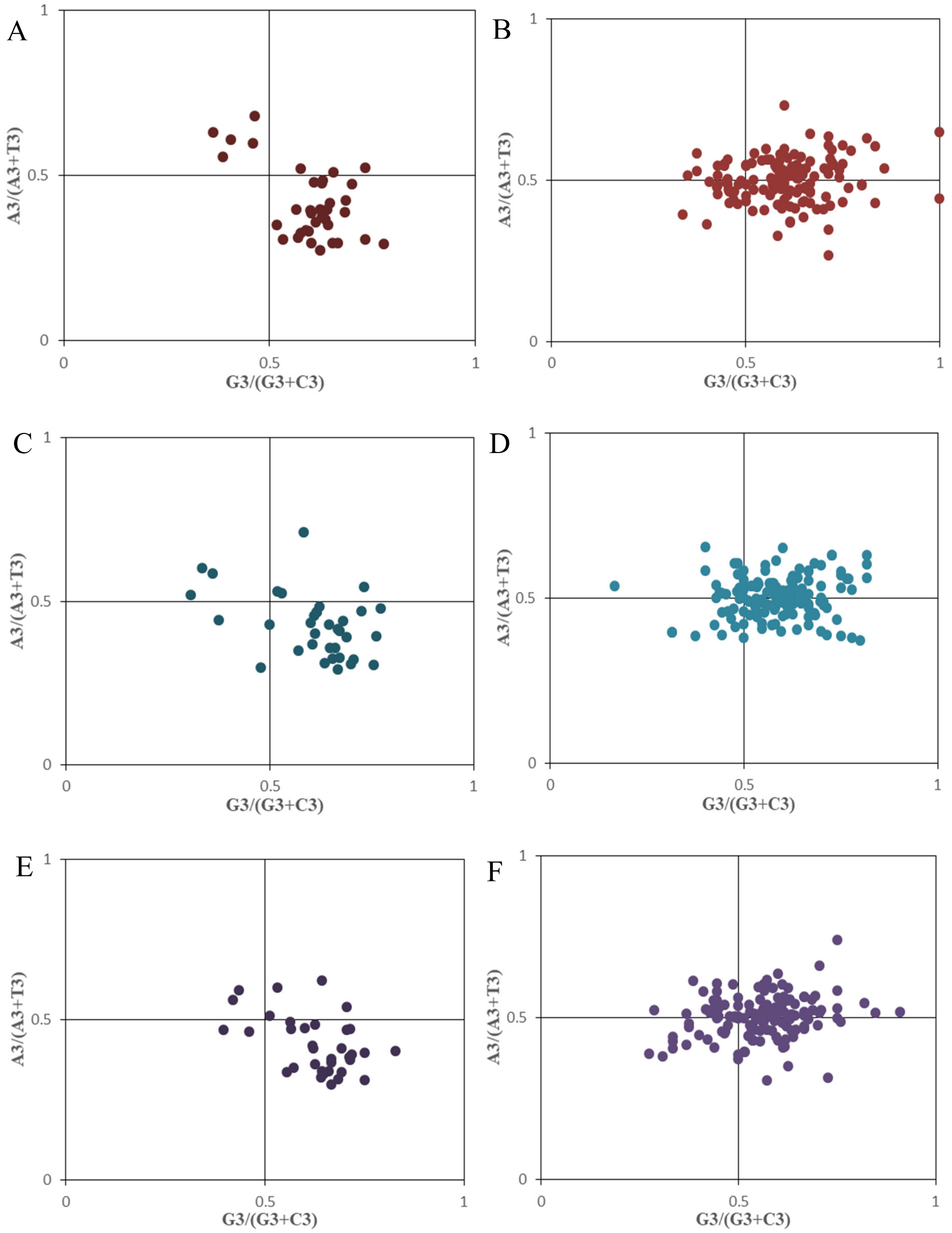

Figure S4. PR2-plot analysis of mtDNAs and cpDNAs in Fucales, Ectocarpales, and Laminariales. (**A**) mtDNA of Fucales, (**B**) cpDNA of Fucales, (**C**) mtDNA of Ectocarpales, (**D**) cpDNA of Ectocarpales, (**E**) mtDNA of Laminariales, and (**F**) cpDNA of Laminariales.
