# Supplementary material for: Organellar Genomes of Sargassum hemiphyllum var. chinense Provide Insight into the Characteristics of Phaeophyceae
Source: Int J Mol Sci. 2024 Aug 6;25(16):8584. doi: 10.3390/ijms25168584 (PMC11354929; doi:10.3390/ijms25168584)
Supplement: Supplementary file 1 [file ijms-25-08584-s001.zip › Figure S5. RSCU analysis chart of mtDNA and cpDNA in Fucales, Ectocarpales and Laminariales.pdf]

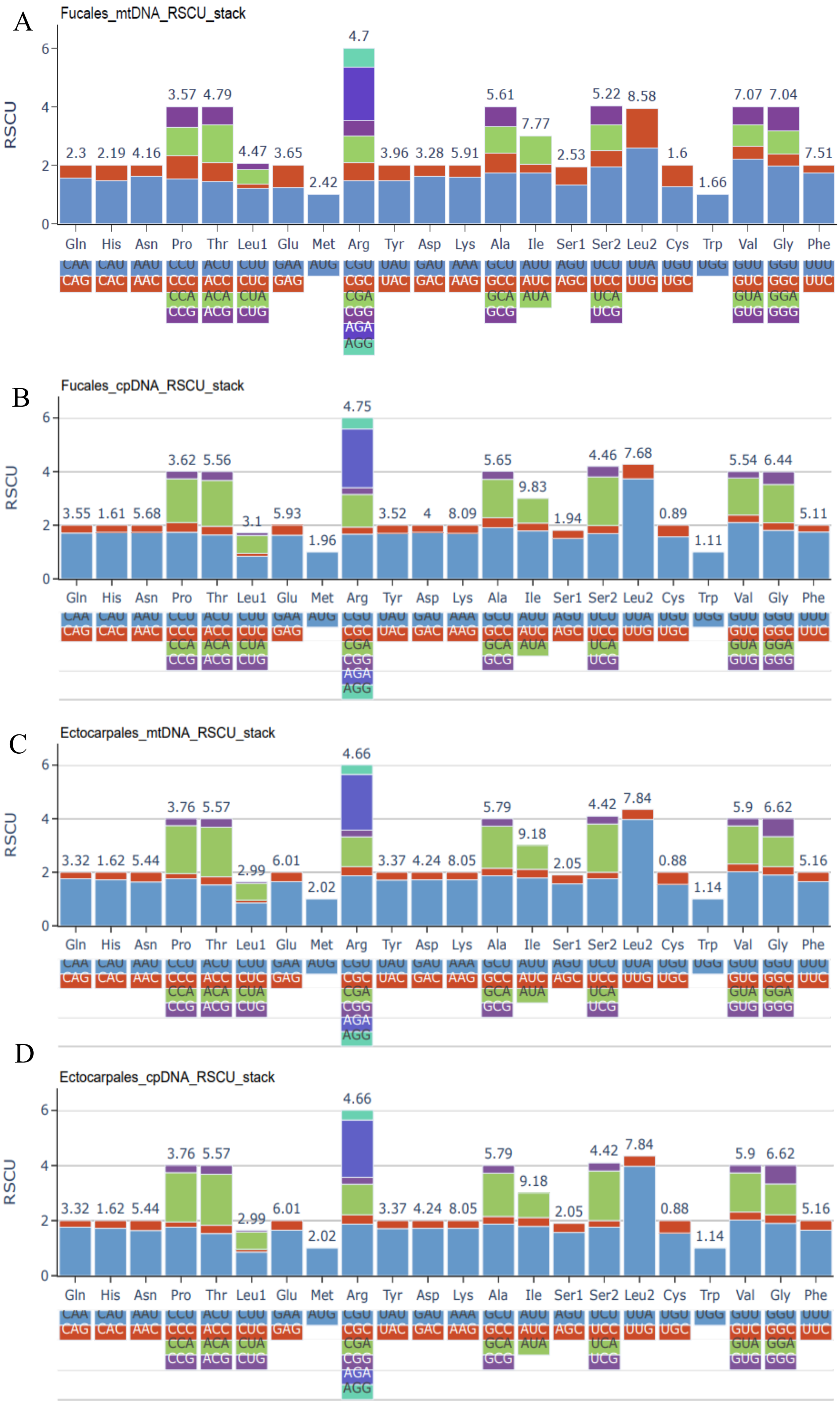
A
Fucales_mtDNA_RSCU_stack
RSCU
0 2 4 6
4.7
2.3 2.19 4.16 3.57 4.79 4.47 3.65 2.42 3.96 3.28 5.91 5.61 7.77 2.53 5.22 8.58 1.6 1.66 7.07 7.04 7.51
Gln His Asn Pro Thr Leu1 Glu Met Arg Tyr Asp Lys Ala Ile Ser1 Ser2 Leu2 Cys Trp Val Gly Phe
CAA CAU AAU CCU ACU CUU GAA AUG CGU UAU GAU AAA GCU AUU AGU UCU UUA UGU UGG GUU GGU UUU
CAG CAC AAC CCC ACC CUC GAG CGC UAC GAC AAG GCC AUC AGC UCC UUG UGC GUC GGC UUC
CCA ACA CUA CGA GCA AUA UCA GUA GGA
CCG ACG CUG CGG GCG UCG GUG GGG
AGA
AGG
B
Fucales_cpDNA_RSCU_stack
RSCU
0 2 4 6
4.75
3.55 1.61 5.68 3.62 5.56 3.1 5.93 1.96 3.52 4 8.09 5.65 9.83 1.94 4.46 7.68 0.89 1.11 5.54 6.44 5.11
Gln His Asn Pro Thr Leu1 Glu Met Arg Tyr Asp Lys Ala Ile Ser1 Ser2 Leu2 Cys Trp Val Gly Phe
CAA CAU AAU CCU ACU CUU GAA AUG CGU UAU GAU AAA GCU AUU AGU UCU UUA UGU UGG GUU GGU UUU
CAG CAC AAC CCC ACC CUC GAG CGC UAC GAC AAG GCC AUC AGC UCC UUG UGC GUC GGC UUC
CCA ACA CUA CGA GCA AUA UCA GUA GGA
CCG ACG CUG CGG GCG UCG GUG GGG
AGA
AGG
C
Ectocarpales_mtDNA_RSCU_stack
RSCU
0 2 4 6
4.66
3.32 1.62 5.44 3.76 5.57 2.99 6.01 2.02 3.37 4.24 8.05 5.79 9.18 2.05 4.42 7.84 0.88 1.14 5.9 6.62 5.16
Gln His Asn Pro Thr Leu1 Glu Met Arg Tyr Asp Lys Ala Ile Ser1 Ser2 Leu2 Cys Trp Val Gly Phe
CAA CAU AAU CCU ACU CUU GAA AUG CGU UAU GAU AAA GCU AUU AGU UCU UUA UGU UGG GUU GGU UUU
CAG CAC AAC CCC ACC CUC GAG CGC UAC GAC AAG GCC AUC AGC UCC UUG UGC GUC GGC UUC
CCA ACA CUA CGA GCA AUA UCA GUA GGA
CCG ACG CUG CGG GCG UCG GUG GGG
AGA
AGG
D
Ectocarpales_cpDNA_RSCU_stack
RSCU
0 2 4 6
4.66
3.32 1.62 5.44 3.76 5.57 2.99 6.01 2.02 3.37 4.24 8.05 5.79 9.18 2.05 4.42 7.84 0.88 1.14 5.9 6.62 5.16
Gln His Asn Pro Thr Leu1 Glu Met Arg Tyr Asp Lys Ala Ile Ser1 Ser2 Leu2 Cys Trp Val Gly Phe
CAA CAU AAU CCU ACU CUU GAA AUG CGU UAU GAU AAA GCU AUU AGU UCU UUA UGU UGG GUU GGU UUU
CAG CAC AAC CCC ACC CUC GAG CGC UAC GAC AAG GCC AUC AGC UCC UUG UGC GUC GGC UUC
CCA ACA CUA CGA GCA AUA UCA GUA GGA
CCG ACG CUG CGG GCG UCG GUG GGG
AGA
AGG

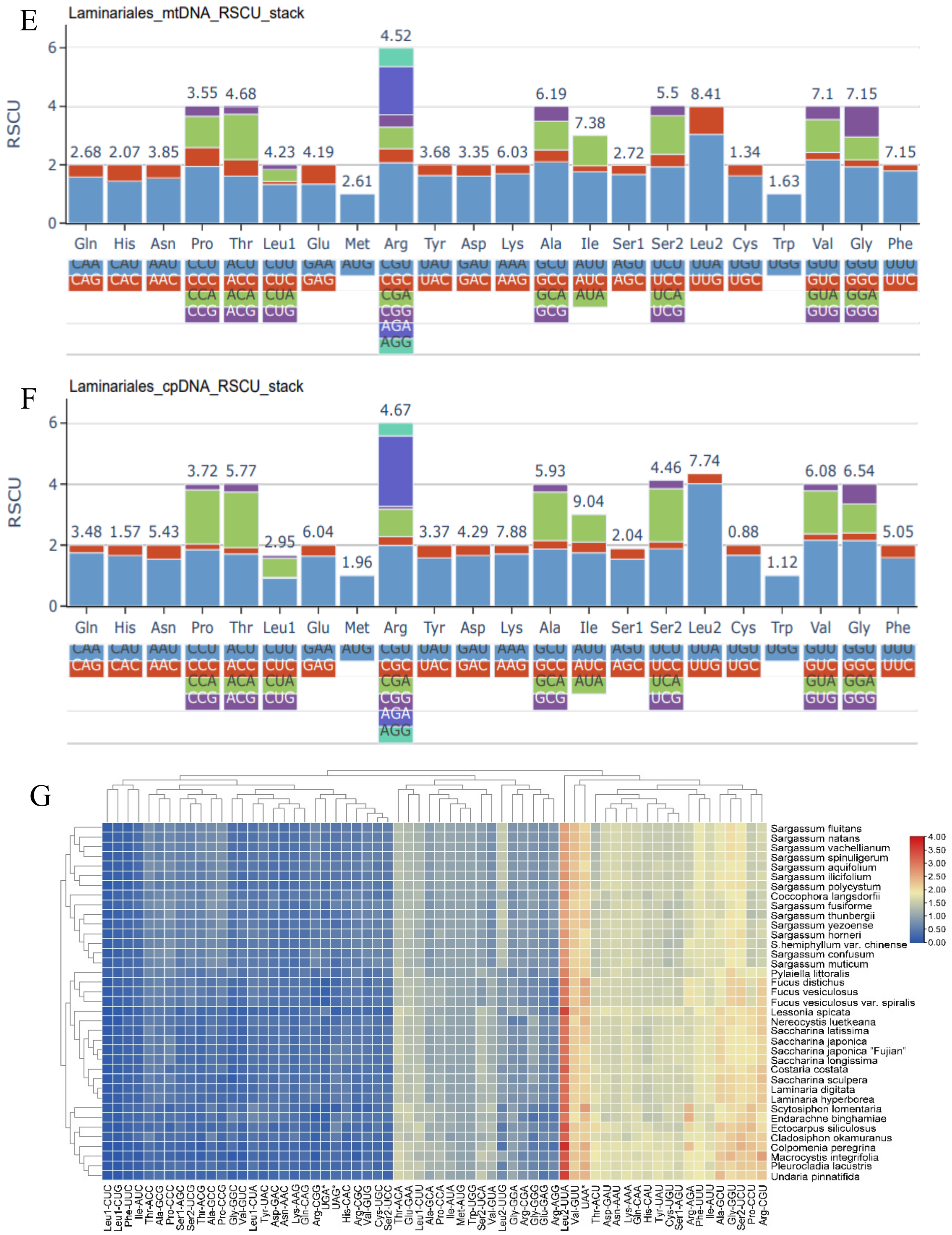

E
Laminariales_mtDNA_RSCU_stack
RSCU
2.68 2.07 3.85 3.55 4.68 4.23 4.19 2.61 4.52 3.68 3.35 6.03 6.19 7.38 2.72 5.5 8.41 1.34 1.63 7.1 7.15 7.15
Gln His Asn Pro Thr Leu1 Glu Met Arg Tyr Asp Lys Ala Ile Ser1 Ser2 Leu2 Cys Trp Val Gly Phe
CAA CAU AAU CCU ACU CUU GAA AUG CGU UAU GAU AAA GCU AUU AGU UCU UUA UGU UGG GUU GGU UUU
CAG CAC AAC CCC ACC CUC GAG CGC UAC GAC AAG GCC AUC AGC UCC UUG UGC GUC GGC UUC
CCA ACA CUA CGA GCA AUA UCA GUA GGA
CCG ACG CUG CGG GCG UCG GUG GGG
AGA
AGG
F
Laminariales_cpDNA_RSCU_stack
RSCU
3.48 1.57 5.43 3.72 5.77 2.95 6.04 1.96 4.67 3.37 4.29 7.88 5.93 9.04 2.04 4.46 7.74 0.88 1.12 6.08 6.54 5.05
Gln His Asn Pro Thr Leu1 Glu Met Arg Tyr Asp Lys Ala Ile Ser1 Ser2 Leu2 Cys Trp Val Gly Phe
G
Sargassum fluitans
Sargassum natans
Sargassum vachellianum
Sargassum spinuligerum
Sargassum aquifolium
Sargassum ilicifolium
Sargassum polycystum
Coccophora langsdorfii
Sargassum fusiforme
Sargassum thunbergii
Sargassum yezoense
Sargassum horneri
S.hemiphyllum var. chinense
Sargassum confusum
Sargassum muticum
Pylaiella littoralis
Fucus distichus
Fucus vesiculosus
Fucus vesiculosus var. spiralis
Lessonia spicata
Nereocystis luetkeana
Saccharina latissima
Saccharina japonica
Saccharina japonica "Fujian"
Saccharina longissima
Costaria costata
Saccharina sculpera
Laminaria digitata
Laminaria hyperborea
Scytosiphon lomentaria
Endarachne binghamiae
Ectocarpus siliculosus
Cladosiphon okamuranus
Colpomenia peregrina
Macrocystis integrifolia
Pleurocladia lacustris
Undaria pinnatifida
4.00 3.50 3.00 2.50 2.00 1.50 1.00 0.50 0.00

H

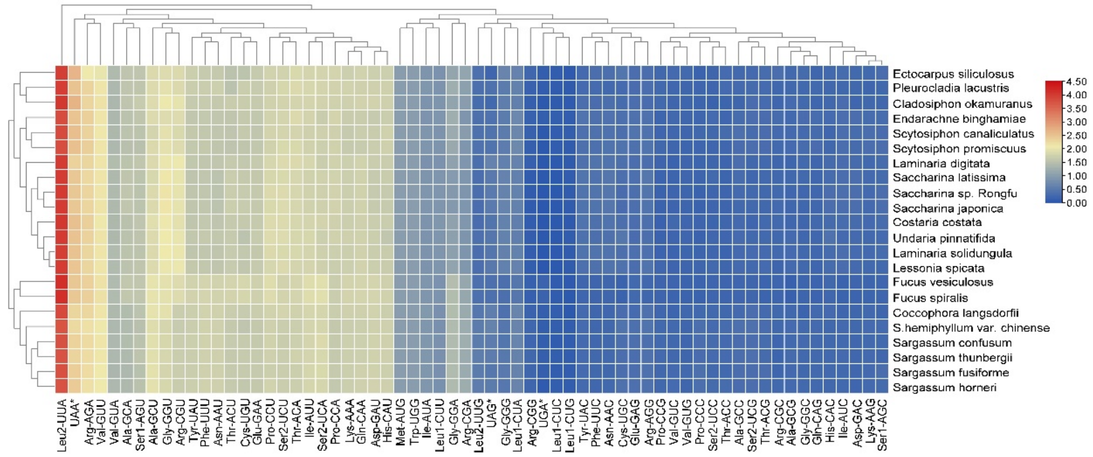

Figure S5. RSCU analysis chart of mtDNAs and cpDNAs in Fucales, Ectocarpales, and Laminariales. RSCU stack diagrams of (**A**) Fucales mtDNA, (**B**) Fucales cpDNA; (**C**) Ectocarpales mtDNA, (**D**) Ectocarpales cpDNA, (**E**) Laminariales mtDNA, and (**F**) Laminariales cpDNA. RSCU value heat maps of (**G**) mtDNA and (**H**) cpDNA.
