# Supplementary material for: Organellar Genomes of Sargassum hemiphyllum var. chinense Provide Insight into the Characteristics of Phaeophyceae
Source: Int J Mol Sci. 2024 Aug 6;25(16):8584. doi: 10.3390/ijms25168584 (PMC11354929; doi:10.3390/ijms25168584)
Supplement: Supplementary file 1 [file ijms-25-08584-s001.zip › Figure S6. Interspersed repeats of mtDNA and cpDNA in the Sargassum hemiphyllum var. chinense.pdf]

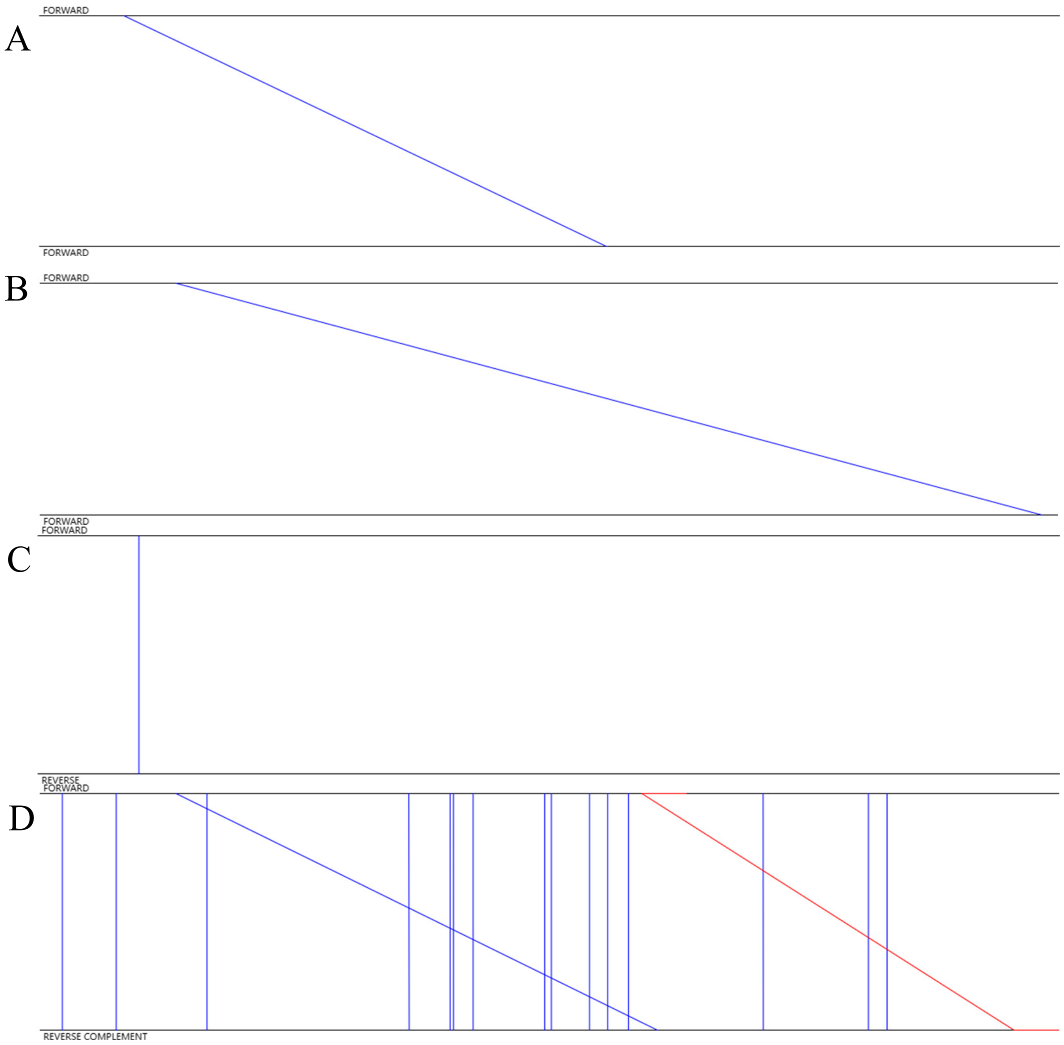

Figure S6. Interspersed repeats of mtDNA and cpDNA of *Sargassum hemiphyllum* var. *chinense*. (**A**) forward repeat of mtDNA, (**B**) forward repeat of cpDNA, (**C**) reverse repeat of cpDNA, and (**D**) palindrome repeat of cpDNA.
