# Supplementary material for: Organellar Genomes of Sargassum hemiphyllum var. chinense Provide Insight into the Characteristics of Phaeophyceae
Source: Int J Mol Sci. 2024 Aug 6;25(16):8584. doi: 10.3390/ijms25168584 (PMC11354929; doi:10.3390/ijms25168584)
Supplement: Supplementary file 1 [file ijms-25-08584-s001.zip › Figure S7. RSCU clustering plot of species mtDNA related to Sargassum hemiphyllum var. chinense.pdf]

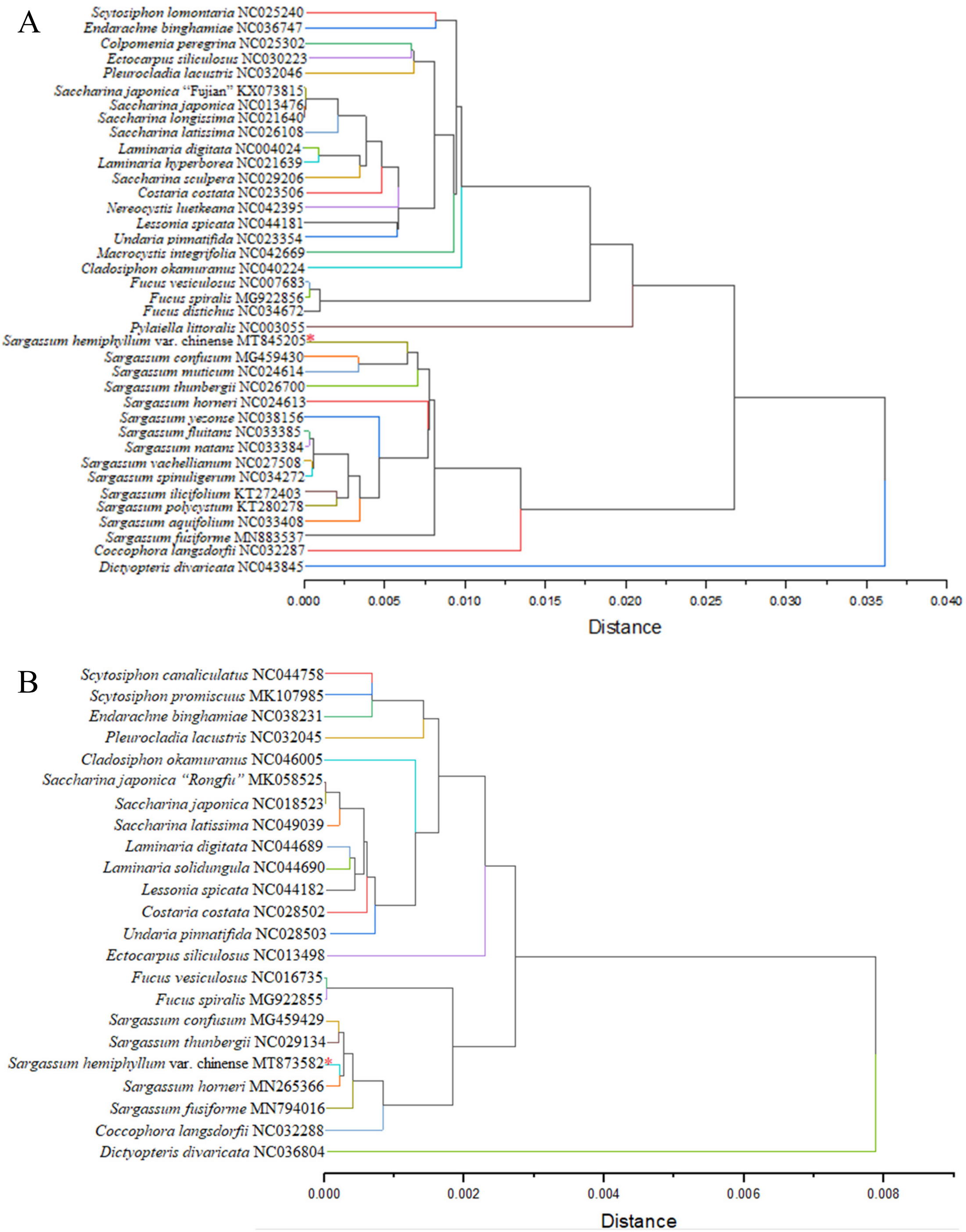

Figure S7. RSCU clustering plot of species (**A**) mtDNAs and (**B**) cpDNAs related to *Sargassum hemiphyllum* var. *chinense*
