# Supplementary material for: Organellar Genomes of Sargassum hemiphyllum var. chinense Provide Insight into the Characteristics of Phaeophyceae
Source: Int J Mol Sci. 2024 Aug 6;25(16):8584. doi: 10.3390/ijms25168584 (PMC11354929; doi:10.3390/ijms25168584)
Supplement: Supplementary file 1 [file ijms-25-08584-s001.zip › Figure S8. Phylogenetic tree (A) mtDNAs and (B) cpDNAs of species related to Sargassum hemiphyllum var. chinense.pdf]

A

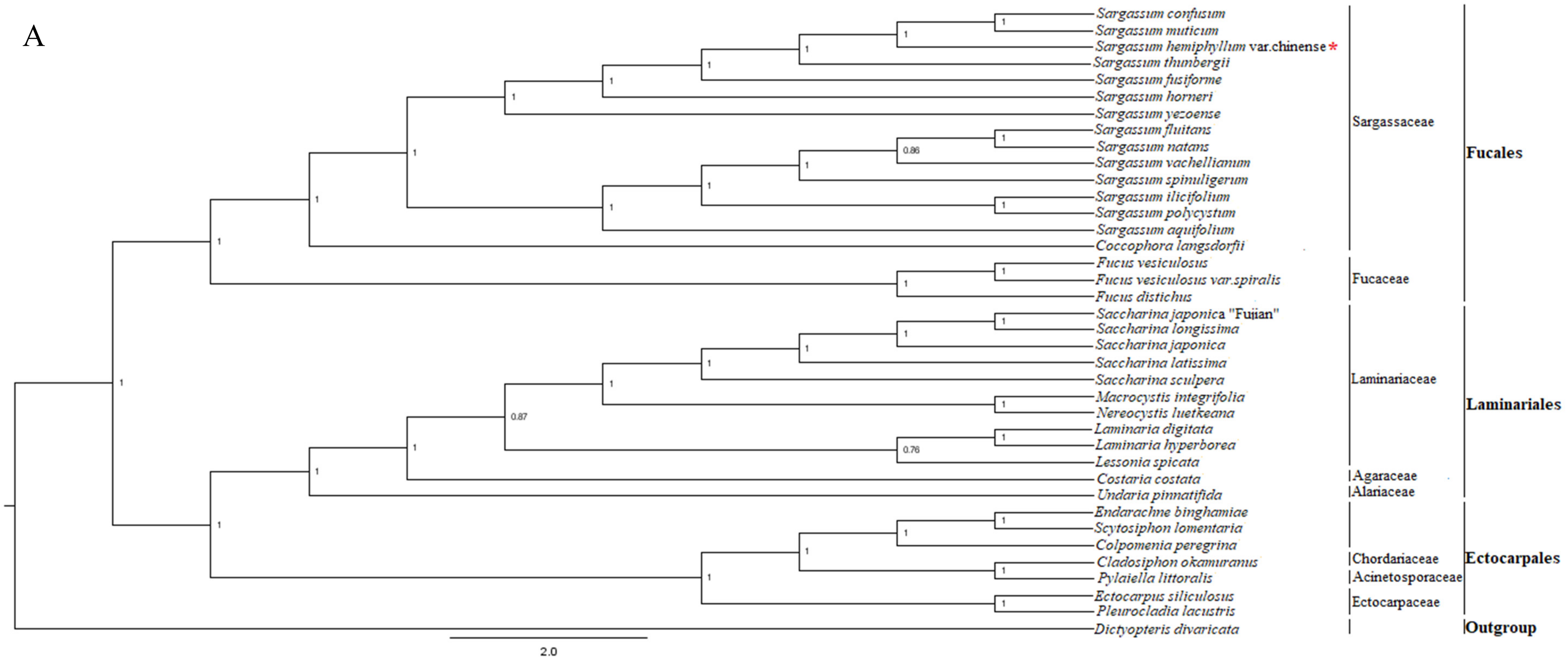

Sargassum confusum
Sargassum muticum
Sargassum hemiphyllum var.chinense *
Sargassum thunbergii
Sargassum fusiforme
Sargassum horneri
Sargassum yezoense
Sargassum fluitans
Sargassum natans
Sargassum vachellianum
Sargassum spinuligerum
Sargassum ilicifolium
Sargassum polycystum
Sargassum aquifolium
Coccophora langsdorfii
Fucus vesiculosus
Fucus vesiculosus var.spiralis
Fucus distichus
Saccharina japonica "Fujian"
Saccharina longissima
Saccharina japonica
Saccharina latissima
Saccharina sculpera
Macrocystis integrifolia
Nereocystis luetkeana
Laminaria digitata
Laminaria hyperborea
Lessonia spicata
Costaria costata
Undaria pinnatifida
Endarachne binghamiae
Scytosiphon lomentaria
Colpomenia peregrina
Cladosiphon okamuranus
Pylaiella littoralis
Ectocarpus siliculosus
Pleurocladia lacustris
Dictyopteris divaricata
Sargassaceae
Fucaceae
Laminariaceae
Agaraceae
Alariaceae
Chordariaceae
Acinetosporaceae
Ectocarpaceae
Fucales
Laminariales
Ectocarpales
Outgroup
0.86
0.87
0.76
2.0

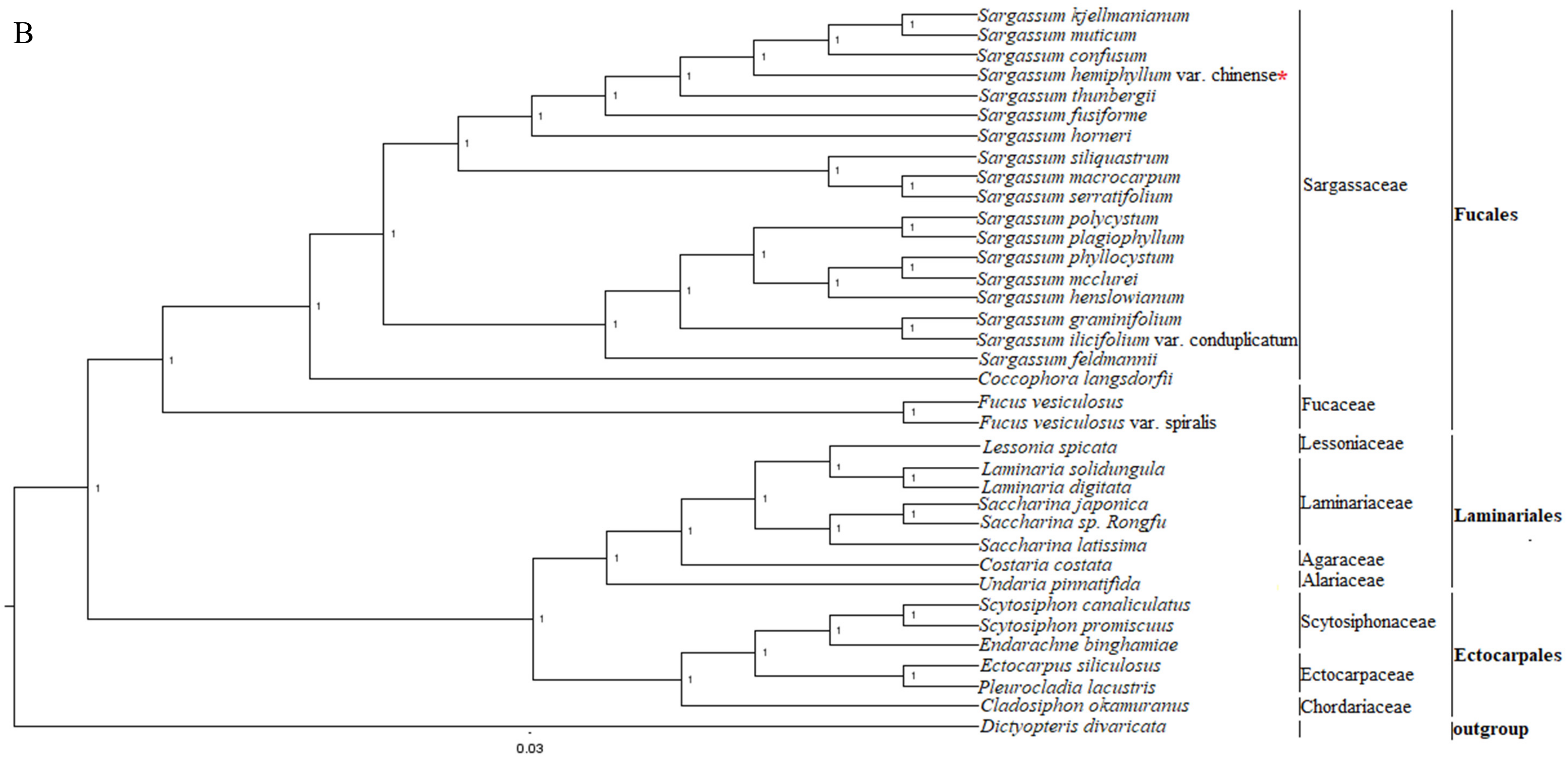

Figure S8. Phylogenetic tree of species (**A**) mtDNAs and (**B**) cpDNAs related to *Sargassum hemiphyllum* var. *chinense*
