# Supplementary material for: Organellar Genomes of Sargassum hemiphyllum var. chinense Provide Insight into the Characteristics of Phaeophyceae
Source: Int J Mol Sci. 2024 Aug 6;25(16):8584. doi: 10.3390/ijms25168584 (PMC11354929; doi:10.3390/ijms25168584)
Supplement: Supplementary file 1 [file ijms-25-08584-s001.zip › Figure S9. Comparison of (A) mtDNAs and (B) cpDNAs from reported species of three orders Fucales, Ectocarpales, and Laminariales.pdf]

A

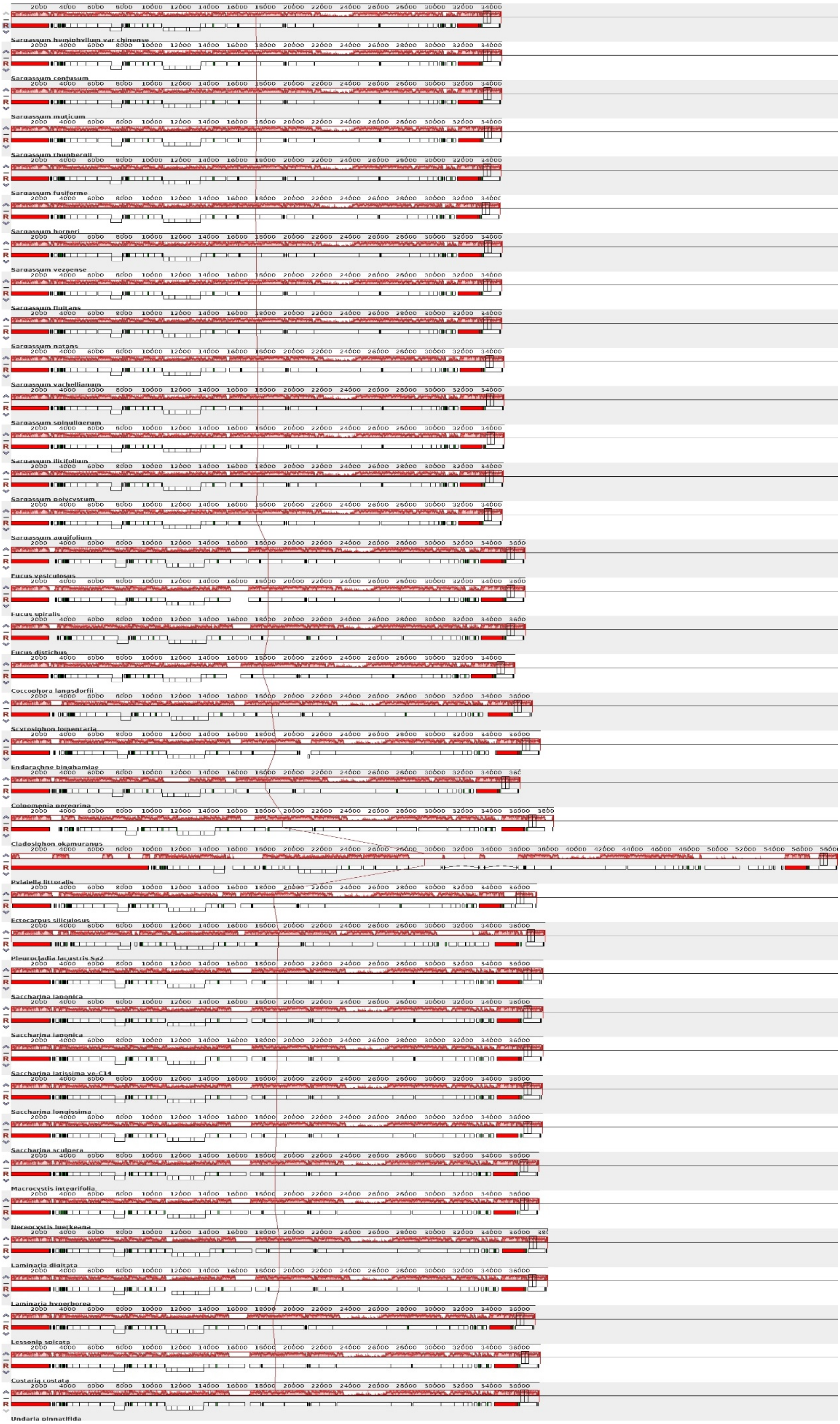
Sargassum hemiphyllum var chinense
Sargassum confusum
Sargassum muticum
Sargassum thunbergii
Sargassum fusiforme
Sargassum horneri
Sargassum yezoense
Sargassum fluitans
Sargassum natans
Sargassum vachellianum
Sargassum spinuligerum
Sargassum ilicifolium
Sargassum polycystum
Sargassum aquifolium
Fucus vesiculosus
Fucus spiralis
Fucus distichus
Coccophora langsdorfii
Scytosiphon lomentaria
Endarachne binghamiae
Colpomenia peregrina
Cladosiphon okamuranus
Pylaiella littoralis
Ectocarpus siliculosus
Pleurocladia lacustris Sa2
Saccharina japonica
Saccharina japonica
Saccharina latissima ye-C14
Saccharina longissima
Saccharina sculpera
Macrocystis integrifolia
Nereocystis luetkeana
Laminaria digitata
Laminaria hyperborea
Lessonia spicata
Costaria costata
Undaria pinnatifida

B

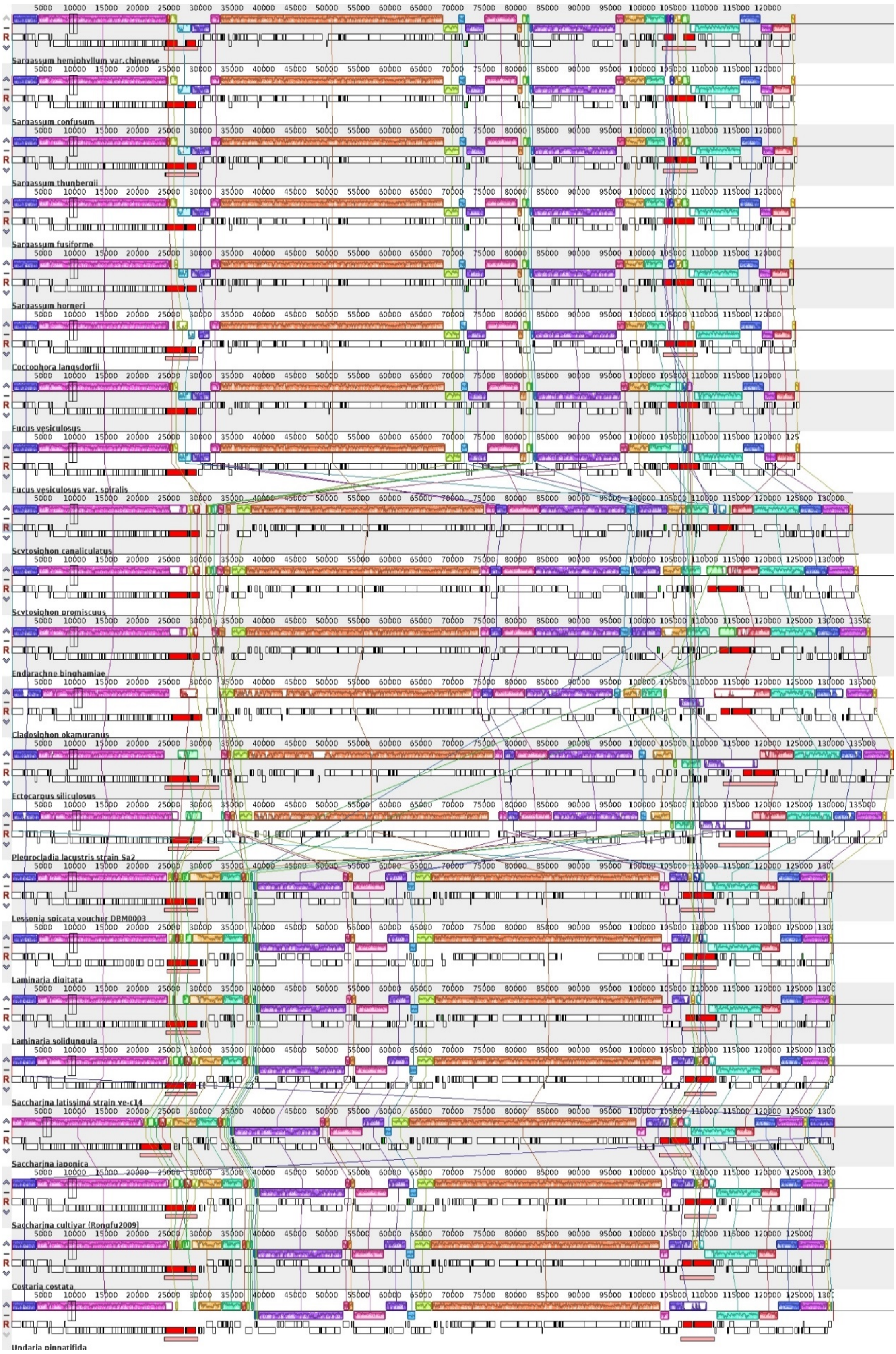

Figure S9. Comparison of (**A**) mtDNAs and (**B**) cpDNAs from reported species of three orders Fucales, Ectocarpales, and Laminariales.
